# Closed-loop fMRI at the mesoscopic scale of columns and layers: Can we do it and why would we want to?

**DOI:** 10.1098/rstb.2023.0085

**Published:** 2024-10-21

**Authors:** Denis Chaimow, Romy Lorenz, Nikolaus Weiskopf

**Affiliations:** ^1^ Department of Neurophysics, Max Planck Institute for Human Cognitive and Brain Sciences, Leipzig, Germany; ^2^ Cognitive Neuroscience & Neurotechnology Group, Max Planck Institute for Biological Cybernetics, Tübingen, Germany; ^3^ Felix Bloch Institute for Solid State Physics, Faculty of Physics and Earth Sciences, Leipzig University, Leipzig, Germany; ^4^ Wellcome Centre for Human Neuroimaging, Institute of Neurology, University College London, 12 Queen Square, London WC1N 3AR, UK

**Keywords:** closed loop, neurofeedback, fMRI, high resolution, cortical columns, cortical layers

## Abstract

Technological advances in fMRI including ultra-high magnetic fields (≥ 7 T) and acquisition methods that increase spatial specificity have paved the way for studies of the human cortex at the scale of layers and columns. This mesoscopic scale promises an improved mechanistic understanding of human cortical function so far only accessible to invasive animal neurophysiology. In recent years, an increasing number of studies have applied such methods to better understand the cortical function in perception and cognition. This future perspective article asks whether closed-loop fMRI studies could equally benefit from these methods to achieve layer and columnar specificity. We outline potential applications and discuss the conceptual and concrete challenges, including data acquisition and volitional control of mesoscopic brain activity. We anticipate an important role of fMRI with mesoscopic resolution for closed-loop fMRI and neurofeedback, yielding new insights into brain function and potentially clinical applications.

This article is part of the theme issue ‘Neurofeedback: new territories and neurocognitive mechanisms of endogenous neuromodulation’.

## 1. Introduction

Real-time fMRI and closed-loop fMRI, particularly fMRI neurofeedback, have become important tools in clinical and cognitive neuroscience [1–4]. Like conventional fMRI studies, closed-loop fMRI relies on relatively low-resolution images that represent the haemodynamic response integrated over typically 2–3 mm large voxels or even larger regions of interest (ROIs). This resolution has proven sufficient to map cognitive processes to brain structures (individual cortical or subcortical areas) as evidenced in a large host of human fMRI literature [5]. Similarly, closed-loop fMRI, neurofeedback and behavioural effects of neurofeedback training were demonstrated at this relatively coarse resolution [1–3]. However, the resolution is not sufficient to study the fine-grained mesoscopic functional organization of the cerebral cortex, i.e. its ‘inner workings’.

**Figure 1.**
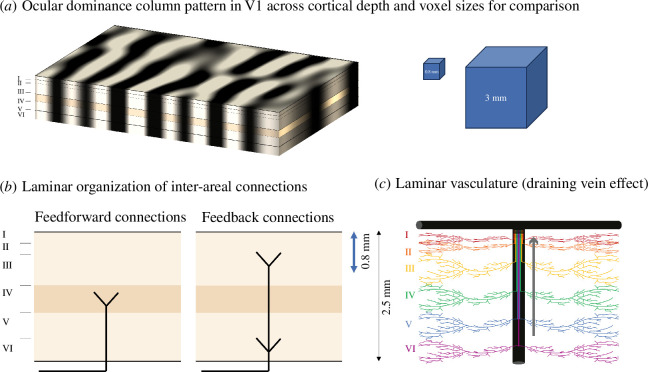
Considerations for mesoscopic fMRI of cortical columns and layers. (*a*) An illustration of an exemplary pattern of ocular dominance columns in primary visual cortex V1 across the cortical sheet and cutting through all cortical layers. A typical voxel at conventional resolution (3 mm) and one currently used in high-resolution fMRI (0.8 mm) are shown for comparison. Sub-millimetre voxel sizes are necessary to begin to resolve different columns and different laminar input and output zones. (*b*) Feedforward input from areas lower in the hierarchy terminates in layer 4 while descending feedback input from higher areas tends to terminate in superficial and deep layers. This proposed scheme allows the partitioning of cortical depth into three main compartments: superficial, middle and deep (modified with permission from [6]). (*c*) Deoxygenated blood is drained towards the surface resulting in reduced spatial specificity, affecting the spatial specificity of conventional BOLD fMRI methods.

The cerebral isocortex is a 2–3 mm thick sheet with six layers and has been shown to be organized into columns in a multitude of brain areas (figure 1). Neurons belonging to the same column, which is typically a few 100 µm wide, respond similarly to certain stimulus features resulting in columnar patterns along the cortical sheet, e.g. ocular dominance and orientation columns in primary visual cortex V1 [7,8]. That is, the mesoscopic columnar structures mediate important cortical functions.

Across the cortical depth, most of the cerebral cortex is organized into six histologically defined layers (figure 1). Neuron types and their connections follow this organization, giving rise to hypothesized cortical microcircuits [9], in which cortical processing is thought to take place. Connections between cortical and subcortical areas also follow the layered organization [10]. For example, ascending feedforward input from the thalamus or other areas that are considered lower in the processing hierarchy terminates in layer 4 while descending feedback input from higher areas tends to terminate in superficial and deep layers [6]. Cortical outputs follow similar laminar patterns. As an example, cortico-spinal outputs from the primary motor cortex predominantly originate in layers 5b and 6 [11].

In non-human primates, invasive electrophysiological studies demonstrated the specific involvement of individual layers during cortical processing, giving rise to mechanistic models of cortical processing [12]. For a long time, neuroimaging in humans could not provide comparable insights because conventional fMRI at 3 T or lower fields lacks the necessary spatial resolution to resolve cortical layers.

To image cortical columnar patterns or different laminar responses, a resolution below 1 mm and preferably closer to 0.5 mm is needed (figure 1*a*). This resolution is still not sufficient to fully resolve each of the six histologically defined layers. However, it is usually assumed that a certain degree of partial volume effect (i.e. a voxel containing signal from more than one tissue type or structure of interest) can be tolerated and dividing the cortical depth into two to three fMRI measurement layers is sufficient, since it provides us with approximate information about different cortical processes. For example, mid-layer feedforward versus top and bottom layer feedback activity [6,13] can be differentiated (figure 1*b*).

Ultra-high magnetic fields (≥7 T), advanced acquisition techniques and advanced data analysis have brought the effective spatial resolution of fMRI into the sub-millimetre mesoscopic range. This made it possible to image cortical column patterns [14–17] and laminar responses in sensory [18,19] and motor cortices [20].

It is important to emphasize that fMRI at the mesoscopic resolution should still be considered a method in development. As will be discussed in more detail below, it faces many methodological and conceptual challenges including the specificity of the BOLD signal with respect to the layer of origin (figure 1*c*). Augmenting this difficulty, cortical thickness varies across regions, with primary motor cortices being the thickest and sensory and association cortices the thinnest [21]. Furthermore, a functional subdivision into feedforward and feedback layers is only one possible model that is most relevant for sensory cortices. For many other cortical areas such functional subdivisions are less well known [22].

Nonetheless, high-resolution fMRI methods are progressing and have recently been used to study higher level cortices [23–25], elucidating layer-specific processes involved in higher level cognition.

Translating the advances of high-resolution fMRI to closed-loop fMRI would allow us to leverage the higher spatial specificity and make the mesoscopic functional cortical architecture accessible to neurofeedback and other applications. For instance, this approach would allow for more precise neurofeedback targets (e.g. cortical columns and layers). Additionally, employing adaptive paradigms and harnessing neurofeedback to induce mesoscopic brain activity could provide valuable insights into cortical functioning at that level. Over time, this may lead to the development of novel clinical interventions targeting mesoscopic neuronal processing.

In this future perspective article, we will explore how a mesoscopic imaging approach may benefit closed-loop fMRI and discuss the challenges that would need to be overcome. While conventional high-resolution fMRI is mainly concerned with acquiring robust signals with high nominal (i.e. sub-millimetre voxel size) and high effective resolution (spatial specificity of the acquired signal), closed-loop fMRI has additional aspects to consider.

Like closed-loop fMRI at standard resolutions, closed-loop fMRI at mesoscopic resolution requires fast and consistent data processing in real time and a high contrast-to-noise ratio for reliable estimates based on single time points or short periods of time. High-resolution acquisitions pose additional challenges to these requirements such as relatively higher noise levels and high data rates. Furthermore, compared to conventional high-resolution fMRI, most closed-loop fMRI applications, particularly neurofeedback, will also require controlling brain activity at that fine spatial granularity. It is unclear to what spatial detail humans can control brain activity, i.e. whether they can achieve control over brain activity at mesoscopic scales.

## 2. Potential benefits of mesoscopic imaging for closed-loop fMRI

We recognize the potential of closed-loop fMRI at the mesoscopic scale across various domains, spanning fundamental neuroscience to clinical applications. This technique promises access to mesoscopic functional units, which play a pivotal role in normal brain function and are also implicated in dysfunctions such as mental health disorders and neurodegeneration. Before discussing specific applications, we will outline how high-resolution fMRI extends the set of neurofeedback targets more generally.

### (a) How mesoscopic fMRI extends the set of possible neurofeedback targets

Generally, there are two ways the higher resolution can benefit neurofeedback approaches. First, for multivariate decoding approaches [26], the higher resolution may provide us with higher information content without explicit reference to any mesoscopic structure. However, increased spatial resolution does not automatically lead to better decoding. There is a trade-off between additional information content and higher noise due to smaller voxels, which is not compensated by the increased number of voxels [27]. So far, the benefit of higher resolution on decoding has been limited [28]. But, the scale at which decoding is optimal depends on the specific decoding task [29] and can vary between regions and individuals [28].

The second way to use this higher resolution is to identify mesoscopic structures like cortical columns or layers within an ROI and average activity over these structures. At first glance, this approach for cortical columns might not be so different from the general decoding approach, such as multivariate pattern analysis (MVPA). Columnar response patterns are mostly functionally defined. That means they first need to be estimated from an independent functional scan before, for example, a feedback signal can be calculated by averaging ongoing signal with respect to that pattern. This can be regarded as the learning stage of a linear multivariate decoder, showing the similarity of MVPA and the averaging approaches.

It has already been demonstrated that stimulus properties coded by sub-millimetre columnar representations can be decoded using much larger voxels at standard field strengths of 3 T [30,31]. However, while the exact origins of the information used by the decoder are subject to debate [32], mesoscopic resolution fMRI allows us to attribute signals precisely to columnar structures and exclude regional and global effects. This would for example open up the possibility to study whether and how the volitional control of columnar activity in sensory cortices influences perception.

The need for high resolution is perhaps even more obvious for closed-loop fMRI studies that target anatomically defined and less distributed structures. This may include deep brain structures that are less accessible using electroencephalography (EEG) but also specific cortical layers. One possibility would be to simply use the average signal of a predetermined range of cortical depths as a neurofeedback target. However, due to spatial correlations caused by sampling and neurovascular signal spread, its response may already be well represented by a response pooled across all layers. A more promising approach would be to calculate spatial contrasts between different layers that would also benefit from implicit denoising (see §4a).

In addition to computing univariate laminar-specific signals and contrasts between layers, it is also possible to apply MVPA in a layer-specific manner. For example, Muckli *et al*. [33] showed layer specificity of decoding of feedforward and feedback information related to visual scenes. Such layer-specific decoding could also be used as a feedback signal in neurofeedback studies.

Laminar responses offer a unique way of inferring directional connectivity between cortical regions as they are expected to reflect feedforward and feedback signals terminating in different layers (figure 1*b*). At conventional resolutions, most connectivity measures used as neurofeedback signals rely on a non-directional correlation of activity between areas [34–37] and are difficult to estimate from limited amounts of data points available in real-time approaches [34,38]. Leveraging the laminar distribution of responses across different areas could potentially supplant or complement these connectivity measures.

Finally, although challenging, it may be worth attempting to target correlational measures of connectivity between layers for neurofeedback. Between-layer connectivity may reflect the current mode of operation within that area. For example, Bastos *et al*. [12] hypothesized that deep to upper layer connectivity controls working memory delay activity in the prefrontal cortex.

### (b) Potential clinical applications of laminar neurofeedback

The application of high-resolution fMRI to explore the role of different cortical layers in neurological and psychiatric disorders has received limited attention, but this is beginning to change [39,40]. Several clinical conditions are associated with laminar-dependent structural and related functional changes. The ability to target laminar responses in neurofeedback could be helpful in discovering therapeutic approaches. Here, we speculate about possible future directions such approaches could take.

A potentially promising area may be the treatment of psychosis symptoms like hallucinations and delusions that are associated with various psychiatric and neurological disorders, including schizophrenia, bipolar disorder, Parkinson’s and Alzheimer’s disease [41]. Predictive coding theories hypothesize that hallucinations and delusions result from aberrant neural signalling of prior beliefs, sensory input and/or prediction errors [42–46], reviewed by Haarsma *et al*. [41]. It is further assumed that prior beliefs are signalled by top–down connections terminating at superficial and deep layers while sensory inputs and prediction errors propagate bottom–up, terminating at middle layers. Therefore, such aberrant neural signalling would be reflected in the changes in laminar activity and could be the target of neurofeedback training.

Related to that are laminar differences between veridical and imagery perception found by layer fMRI studies in healthy subjects [47–49]. Based on their results, Carricarte *et al*. [47] speculate that this layer segregation may be disturbed by hallucinations.

Other conditions that are being addressed by theories of predictive coding and hierarchical concepts are autism [50,51], depression [52,53] and psychosomatic disorders including pain and placebo effects [54,55]; for a review see [54].

Neurodegenerative diseases such as Huntington’s disease also show layer- and connectivity-specific pathological patterns and disease progression [56], which may be more directly targeted with laminar-specific closed-loop approaches than current ROI approaches [57]. While speculative, such structural and functional alterations may, in principle, motivate neurofeedback studies targeting these or other compensatory structures.

### (c) Closed-loop laminar paradigms to study cortical functioning

One important aspect of closed-loop approaches is that they can reverse the traditional paradigm of an fMRI study, where brain activity is studied depending on the experimental paradigm, e.g. behaviour and/or task stimulation. Controlling brain activity in specific ways, e.g. after neurofeedback learning, allows us to consider brain activity as the independent and behaviour as the dependent variable. In other words, it may be considered as a form of ‘physiological’ brain stimulation approach facilitating causal inferences. Unlike conventional stimulation methods such as transcranial magnetic stimulation (TMS), targets can be chosen across the entire brain and complex activity patterns may be achieved.

Thus, a closed-loop fMRI and neurofeedback approach may help improve our understanding of the functional role different layers play in human high-level cognition. To date, little data exist on the laminar functional specificity of a given brain region that could guide experimental design in layer fMRI studies; this is especially true for high-level association cortices. So far, many layer fMRI studies are mainly confirmatory or often feasibility studies that rely on relatively simple models of laminar involvement [22]. These models are mostly based on hypothesized bottom–up feedforward (in the middle layer) and top–down feedback (in the superficial and deep layers) activations in sensory cortices during simple perception tasks. If results deviate from the expected hypothesis, the relatively low confidence in the still maturing layer fMRI methods often hampers the consideration of alternative explanations and revision of the mechanistic explanation.

We anticipate that successful closed-loop fMRI and neurofeedback approaches will enhance the robustness of layer fMRI results and help us to go beyond confirmatory studies. For example, if high-resolution neurofeedback succeeds in inducing specific laminar activity patterns, an alternative approach may be used to probe behavioural effects. Given a task that an area is known to be involved in, one could study the specific effects that up- and downregulations of superficial and deep layers within that area would have on task performance. Neurofeedback effects on task performance due to volitional regulation of activity in specific cortical regions have been shown for visual [58], motor [59,60], language [61], memory [60], pain perception [62] and emotion [63,64] tasks.

Similarly, it is possible to leverage closed-loop fMRI approaches to identify the exact cognitive task conditions that selectively activate layers in ROIs or networks of interest (e.g. superficial versus deep layer dissociation). Lorenz *et al*. [65] have developed and validated neuroadaptive Bayesian optimization, a closed-loop approach that combines real-time fMRI and machine learning. This approach involves an automatic and intelligent search across a large cognitive task space, with fMRI data analysed in real time and the subsequent task condition or stimulus chosen based on the real-time results. The approach has been successfully employed to better understand the functional role of frontoparietal networks in healthy individuals [66] and map cognitive dysfunction in frontoparietal brain networks in aphasic stroke patients [67].

Importantly, in the future, this method could be used in combination with high-resolution fMRI to characterize laminar response profiles throughout the brain. Unlike conventional layer fMRI studies, it does not rely on selecting a single or a few cognitive tasks in a relatively ad hoc manner speculating that this will lead to a desired laminar dissociation. Closed-loop fMRI at ultra-high-resolution provides a powerful strategy to efficiently explore many more task conditions than currently possible with standard methodology in a single fMRI scan [68]. Thus, it may more quickly and comprehensively advance our knowledge of how different layers contribute to higher level human cognition.

### (d) Studying mechanisms and effects of neurofeedback

This article is mainly concerned with using high-resolution fMRI directly in a closed-loop setting extracting information online. However, high-resolution functional and structural MRI can also be used separately and offline to study the mechanisms and effects of neurofeedback.

Neurofeedback training is associated with changes in functional activity [69]. For example, Shibata *et al*. [69] demonstrated that implicit neurofeedback training resulted in biases of ongoing activity with respect to patterns representing a specific stimulus orientation and had effects on perceptual discrimination. Does such perceptual learning affect the activity of all layers equally? High-resolution fMRI could allow the characterization of such functional changes in a layer-specific manner.

High-resolution fMRI may be used to study localized effects of EEG neurofeedback, similar to previous multi-modal EEG-fMRI studies at lower resolution [70]. For example, high-resolution fMRI may provide new insights into the modulation of brain rhythms. Modulation of alpha, beta and gamma band activity correlated with the BOLD signal at different cortical depths [71,72], in line with different roles in feedforward and feedback communication between regions.

In addition to functional changes, neurofeedback training has been shown to result in structural changes [57,73]. The brain’s microstructure including myelin, iron and neuronal fibres can be imaged *in vivo* at high resolutions [74,75]. These methods are also being applied to laminar organization [76], which allow us to study subtle changes in the cortical microstructure due to pathological processes but also in development and training-induced plasticity [77]. For example, Whitaker *et al*. [78] used magnetization transfer (MT) saturation across cortical depth and cortical thickness measurements to track intracortical maturation and myelination during development. Importantly, recent developments in quantitative MRI promise to characterize the nature of plasticity-induced changes by combining multiple complementary quantitative MRI maps [79] together with biophysical modelling [75].

## 3. Current state of ultra-high-field high-resolution real-time fMRI

Having explored the vast possible applications of mesoscopic closed-loop fMRI, we shift our focus to the current state of ultra-high-field high-resolution real-time fMRI. So far, only a few real-time fMRI studies have used ultra-high fields and even fewer used sub-millimetre resolution or specifically targeted structures at the mesoscopic scale. A number of studies are related to brain-computer–interfaces (BCI) [80], which aim to use brain activity acquired in real time to control an external device, either with or without feedback.

Andersson *et al*. [81,82] decoded the locus of covert attention in real time from 7 T fMRI data using a resolution of 1.84 × 1.84 × 2 mm^3^ voxels. Interested in invasive BCI applications, their goal was to demonstrate the potential of 7 T fMRI to identify cortical target regions and to train patients before undergoing electrocorticogram (ECoG) implantation. In another BCI motivated study, Kaas *et al*. [83] demonstrated the possibility of decoding somatosensory imagery in real time from 7 T data using 1.5 mm isotropic resolution. These studies did not target the mesoscopic sub-millimetre scale, rather they used 7 T to benefit from its higher signal-to-noise ratio for decoding (SNR) [83] or as an ECoG surrogate.

A notable exception is a proof-of-concept study [84], in which imagined letters were decoded via real-time reconstruction from the early visual cortex and fed back to the subject. While not directly targeting columns or layers, this study used 0.8 mm isotropic resolution to better resolve the retinotopic activity patterns in the early visual cortex. Though no comparison to lower resolution or data acquired at 3 T has been made, such studies are likely to benefit from higher resolution and the smaller spatial spread of retinotopically represented details at 7 T [85].

High-resolution acquisitions or ultra-high field MRI is used even more rarely for fMRI *neurofeedback*. Gröne *et al*. [86] performed a neurofeedback study at 3 T and 7 T using 3 mm isotropic resolution. Participants had to learn to control their brain activity in the anterior cingulate cortex (ACC). They found a higher success rate of ACC activation in the 3 T compared with the 7 T group. Interestingly, in this study, 3 T even gave a higher temporal SNR (tSNR, defined as the mean signal level divided by the standard deviation of noise over time) than 7 T . These results are somewhat surprising as 7 T is generally expected to outperform 3 T in terms of SNR [87]. However, at the relatively coarse resolution employed here, SNR gains due to the higher field may have been small and other factors like ultra-high-field-specific artefacts [88] may have played a more prominent role.

We can only speculate about the exact reasons for this finding but it may be related to specific issues of 7 T fMRI, including artefacts specific to ultra-high fields [88] and that it may not be superior in all use cases, e.g. at lower resolutions SNR is more affected by physiological noise (and not thermal noise, see §4a) [87,89] which increases with field strength approximately proportional to the signal.

A slightly higher resolution (2 mm isotropic) was used by Russo *et al*. [90] who developed and demonstrated the concept of multi-dimensional feedback, representing points in a representational space of imagined objects, defined by representational similarity analysis.

Lastly, Rasmijn [91] broke ground with the first attempt to combine layer fMRI (analysed offline) and neurofeedback. Participants engaged in two different tasks, namely mental imagery and mental calculation, attempting to control their average BOLD signal in the supplementary motor area (SMA), which was fed back to the participants (i.e. the feedback was not layer specific). After the experiment, laminar differences in the induced activation patterns were analysed offline. While both tasks activated different parts of SMA, there was no clear laminar differentiation as both tasks more strongly activated superficial layers of the supplementary motor area.

This informal literature review shows that while some valuable experience with real-time fMRI at ultra-high fields has been gained, closed-loop fMRI studies that target mesoscopic structures are still lacking.

## 4. Is closed-loop fMRI and neurofeedback possible at the mesoscopic scale?

So far, the feasibility of closed-loop fMRI and neurofeedback at the mesoscopic scale has not been demonstrated. We believe it is largely determined by the technical aspects of acquiring high-resolution fMRI data in real time and the neurobiology underlying mesoscopic brain activity. In the following, we will elaborate on this assessment and lay out the challenges in each domain, propose potential solutions and attempt to gauge the feasibility.

### (a) Technical challenges and solutions for high-resolution fMRI in real time

#### (i) High-resolution fMRI at ultra-high fields

**Figure 2.**
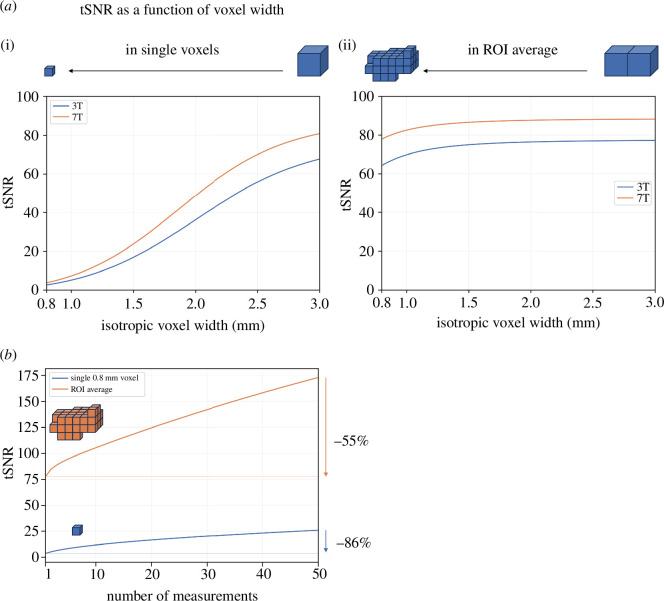
SNR in high-resolution real-time fMRI. (*a*) The effect of increasing spatial resolution on temporal SNR (tSNR, defined as the mean signal level divided by the standard deviation of noise over time) taking into account thermal and physiological noise. (i) Expected tSNR in individual voxels using 3 T and 7 T acquisitions as a function of voxel width. (ii) The tSNR, but this time after averaging all voxels within a constant volume of 980 mm^3^ (equal to that of approx. 36 isotropic 3 mm voxels, which is comparable to ROIs used in neurofeedback studies (e.g. [95]) after splitting the total volume into two layers). This type of averaging is typically performed in laminar fMRI when observing layer response profiles. (*b*) The effect of averaging multiple time points on tSNR. Expected tSNR is shown as a function of averaging a number of consecutive measurements. The blue curve shows tSNR in individual isotropic 0.8 mm voxels (7 T). The tSNR drops by 86% when going from 50 measurements to a single measurement. The orange curve shows the same but for an ROI average of approximately 1914 isotropic 0.8 mm voxels (980 mm^3^ volume, equal to that of approx. 36 isotropic 3 mm voxels). Here, the tSNR drop when going from 50 measurements to a single measurement is more moderate (55%).

Sub-millimetre resolution fMRI relies on ultra-high magnetic fields. The reasons are twofold. First, higher magnetic fields result in higher SNR [92] and higher BOLD responses [93], which together determine the contrast-to-noise ratio (CNR)—a measure of the detectability and reliability of the observed BOLD response in relation to background noise. Noise in fMRI can be approximately partitioned into thermal and physiological noise [87,94]. Thermal noise refers to fluctuations in signal intensity caused by the thermal molecular motion within the subject and receiver electronics and is additive to the signal (and independent of the signal amplitude). Physiological noise is proportional to the signal amplitude and includes BOLD fluctuations but also cardio-respiratory activity and head motion. Signal-to-thermal noise is approximately proportional to the voxel volume and, therefore, effectively limits the achievable resolution (figure 2*a*(i)). The higher SNR and CNR of ultra-high magnetic fields allow us to retain functional sensitivity while increasing the spatial resolution, i.e. the intrinsically higher sensitivity at higher fields can be traded for higher spatial resolution.

Second, the effective resolution of fMRI is not only determined by the voxel size but also by the underlying physiological mechanism of the BOLD signal, which can lead to spatial blurring and imprecisions. Here, ultra-high magnetic fields also increase spatial specificity as the signal becomes more influenced and even dominated by smaller blood vessels that better reflect local neuronal activity [16,85,96].

Although ultra-high fields facilitate high-resolution fMRI in these two important ways, they do not completely alleviate the limitations concerning spatial specificity and sensitivity.

Blood flow in the brain is physiologically controlled at the sub-millimetre scale [97] but resulting changes in the deoxyhemoglobin content are ultimately diluted downstream because draining veins (figure 1*c*) pool blood not only from active but also from non-active regions [98]. Even at ultra-high magnetic fields using standard gradient-echo (GE) BOLD sequences, the signal is still dominated by larger veins, which is a particular problem in laminar imaging as venous blood from deeper layers is being drained towards the surface, displacing and blurring signals originating from different layers (see figure 1*c*) [99]. Alternative acquisition techniques with higher spatial specificity have been used for high-resolution fMRI like spin-echo (SE) BOLD [100] and three-dimensional gradient echo and spin echo (3D-GRASE) [101] that cancel some of the signal caused by larger blood vessels, or vascular space occupancy (VASO), which is sensitive to spatially more specific changes in blood volume [102,103].

Furthermore, ultra-high-field and high-resolution acquisitions bring additional challenges of increased spatial inhomogeneities and higher levels of artefacts and distortions [104]. The requirement for a precise high-resolution signal amplifies the effects of subject motion and the dependence on high-quality registrations and processing [88].

Finally, while laminar fMRI is increasingly being applied to basic cognitive neuroscience questions, it is far from being an established routine method. Its challenges include the influence of vasculature organization, analysis and interpretation with respect to underlying cortical circuit models and higher demands on spatial precision [22]. Furthermore, for layer fMRI to become a standard cognitive neuroscience method, automated and robust analysis routines including objective ROI selection are needed [105].

#### (ii) Is real-time fMRI sufficiently sensitive at the mesoscopic scale?

Real-time fMRI and closed-loop fMRI demand particularly high sensitivity since they typically have to rely on a very limited amount of data acquired almost instantaneously.

This requirement goes against the reduction of SNR when decreasing voxel sizes for high-resolution fMRI. Hence, one may be particularly concerned that the sensitivity of high-resolution fMRI will not be sufficient for closed-loop fMRI. To estimate the feasibility, we theoretically assess how high-resolution fMRI at ultra-high fields would compare to conventional resolution fMRI at 3 T, which was successfully used for various closed-loop fMRI experiments.

Figure 2*a*(i) shows how the temporal signal-to-noise ratio (tSNR) drops when going from 3 mm to 0.8 mm voxel size taking into account thermal and physiological noise, even when at the same time considering the use of higher magnetic field strengths.

However, this is the tSNR in individual voxels. In most closed-loop fMRI applications, we are interested in an aggregate signal, e.g. an average over voxels belonging to a specific layer within an ROI. While such a layer may only be a voxel deep, it would likely still contain many more voxels extending parallel to the cortical sheet. Averaging voxels reduces thermal noise, thereby increasing the relative contribution of physiological noise. Physiological noise, however, is autocorrelated in space and time and depends much less on spatial resolution. Koopmans *et al*. [106] showed that averaging laminar signals over 100 voxels or more shifts the signal into the physiological noise regime. Figure 2*a*(ii) shows that when considering a constant ROI volume, the temporal SNR of the spatially averaged signal shows a much more modest drop when going from 3 mm to 0.8 mm voxel size. This drop can even be largely compensated by the higher field strength.

In addition, larger voxels may include a mixture of different tissue types (partial voluming). Reduced partial voluming in smaller voxels also reduces unwanted contributions of high physiological noise regions at the pial surface to signal sampled from the grey matter. Furthermore, it may reduce the mixing of activated and non-activated brain regions. In both cases, smaller voxel sizes would lead to a higher CNR and functional sensitivity.

From an SNR perspective, the statistical power of a high-resolution real-time acquisition at 7 T after structure-specific averaging may be not that different from that of current standard resolution real-time fMRI at 3 T, which has successfully been used for closed-loop fMRI. The theoretical estimate and feasibility assessment are further supported by studies that found gains in tSNR by acquiring data at a higher resolution followed by smoothing to a lower resolution compared with direct acquisition at the lower resolution [107], especially when the smoothing was anatomically informed [108].

To estimate the impact of having a limited number of time points of sub-millimetre fMRI data available for real-time analysis, we compare the SNR of signals averaged across different numbers of measurements. A conventional non-real-time analysis would average all time points belonging to one condition (e.g. 50) from an entire run. The blue curve in figure 2*b* shows the drop of tSNR in a single voxel when going from 50 to a single measurement (86%). Again, when considering the average of all voxels belonging to a mesoscopic structure (orange curve) the drop becomes much more moderate (55%). The reason for that is the higher relative contribution of temporally autocorrelated physiological noise. Additional factors like temporal instabilities and subject movement, especially at high resolutions, further contribute to diminishing the relative advantage of longer acquisitions.

However, the reliance on a single time point is not a strict requirement for closed-loop fMRI experiments. Frequently, real-time fMRI studies temporally filter and average several time points to increase the statistical power by sliding window (e.g. as often done in neurofeedback) or block-wise averaging (e.g. as in neuroadaptive Bayesian optimization [66]). The experimental design largely determines how many data points enter the closed-loop fMRI computations. In the context of neurofeedback studies, it has been a subject of debate whether continuous feedback [95], i.e. the update of feedback signals after every acquired volume, is necessary or even optimal. An alternative method, intermittent feedback [95], averages acquired signals over an entire block of multiple measurements before presenting the feedback to the participant. This approach would result in further increases in statistical power as shown in figure 2*b*. It also has the added advantage that feedback cannot interfere with other tasks that a participant might be engaged in. In some cases, intermittent feedback has been shown to outperform continuous feedback not only within specific application scenarios [109] but also overall [110,111]. Note, however, that these studies used a sliding-window continuous feedback or temporal low-pass filtering of the neurofeedback signal, resulting in an imperfect comparison, since the single time point condition was not strictly represented.

#### (iii) Techniques to improve sensitivity and their applicability to real-time fMRI

Still, SNR remains a critical factor for high-resolution fMRI and for closed-loop applications like neurofeedback whose effectiveness, i.e. contingency of the feedback [4], depends on robust signal estimates. To this end, data processing methods can help reduce noise levels. That may include real-time use of motion regressors and scrubbing of outlier volumes [38,67], physiological noise reduction methods [112] based on physiological recordings (particularly pulse and respiration), real-time ICA [113], global signal regression or the use of differential feedback that cancels out common noise components [95]. Recently, an offline method to reduce thermal noise contributions has been proposed [114] but it needs to be seen whether it can be adapted to work in a real-time setting. On the acquisition side, multi-echo echo-planar imaging (EPI) [95,115] may be helpful for separating noise from signal sources [116]. However, the need to acquire data from multiple echoes conflicts with the requirement of acquiring many k-space lines within a short window of time in order to achieve high resolutions. An isotropic resolution of 1.6 mm has been demonstrated with a standard 7 T setup [117], but to achieve further gains in resolution stronger gradients and better RF coils may be needed. Using such a set-up, an isotropic resolution of 1.16 mm has been achieved recently [118]. Furthermore, a deep-learning reconstruction algorithm has been proposed as a method to achieve higher resolutions in multi-echo acquisitions [119].

Finally, high-resolution fMRI and layer fMRI, in particular, are affected by spatially varying biases due to MRI instrumentation, including coil sensitivity and field inhomogeneity, as well as anatomical and physiological sources, like vascular anatomy, cortical folding patterns and cortical orientation with respect to the magnetic field [120]. For example, the local presence of larger blood vessels is likely to cause higher BOLD responses, and the orientation of these vessels with respect to the magnetic field is also known to affect the amplitude of the BOLD signal [121]. Furthermore, as mentioned above, high-resolution fMRI is particularly sensitive to temporal instabilities due to acquisition hardware or subject motion, which may be reduced by advanced prospective motion correction [122]. These sources add to variance over space and time.

A better understanding of all variance components and their covariance structures involved in high-resolution and layer fMRI would generally enhance our understanding of specific SNR limits in conventional and real-time layer fMRI. It may also allow us to account for some of these factors by modelling their influences [123] and thereby help us to further increase statistical power.

#### (iv) Techniques to improve spatial specificity and resolution and their applicability to real-time fMRI

Conventional closed-loop fMRI almost exclusively uses GE BOLD fMRI, which suffers from poor spatial specificity due to signal biases stemming from draining veins. Are spatially more specific acquisition techniques like VASO [102,103] or 3D GRASE [101] a viable alternative for closed-loop fMRI? Unfortunately, both methods suffer from considerably diminished sensitivity. Whether this is outweighed by higher specificity is an open question. However, very low contrast-to-noise may be especially problematic for closed-loop fMRI. Furthermore, 3D GRASE and especially VASO cannot achieve the same temporal resolutions as comparable GE BOLD acquisitions, which may further limit their applicability for computing feedback signals with minimal delay.

In our opinion, it may be useful to continue exploring alternative non-GE BOLD acquisition methods for closed-loop high-resolution fMRI also while considering their sensitivity and temporal resolution disadvantages. But preference for any acquisition method will also depend on the specific application. For example, using intermittent feedback, the acquisition speed may not be as critical. On the other hand, analysis for information (e.g. MVPA) may be less sensitive to draining vein biases than response amplitude [33,49,124].

An alternative approach to increase spatial specificity is to correct for draining vein biases in analysis. This can be achieved by fitting biophysical models of laminar BOLD responses (Havlicek and Uludağ, 2020; Heinzle *et al*., 2016; [99,125,126]) to data and extracting parameters describing the underlying neuronal response. This way *a priori* knowledge about the generation of laminar BOLD signals could be optimally used to increase specificity and sensitivity. The application of such models would require additional processing depending on the specifics of the model. Also, additional structural or functional data acquisitions may be needed to estimate model parameters describing the local vasculature and acquisition process. Once such parameters have been estimated, the real-time application would likely be computationally much simpler. It could for example consist of linear deconvolution equations [99].

For real-time layer fMRI, it may be beneficial to sacrifice coverage and resolution in one or two spatial dimensions to gain temporal and nominal cortical depth resolution. Note that since the physiological signal bias remains the same, the overall increase in spatial specificity is lower than the decrease in voxel size. Such approaches include the use of anisotropic voxels [127] or a technique known as fMRI line-scanning [128,129], where within a very narrow coverage volume only one spatial dimension is encoded. The latter has recently been combined with multi-echo acquisitions, prospective motion correction and non-real-time compatible thermal denoising [130].

#### (v) Data processing

High-resolution fMRI places additional conceptual and technical requirements on data processing. When targeting mesoscopic structures, precise anatomical reference information is needed to extract signals with respect to structures of interest like cortical columns and layers. A separate session may be needed to acquire the necessary data which are then processed in advance. Such processing may include segmentation and estimation of cortical depths [131]. During the real-time fMRI session, this reference data must be registered quickly and precisely to ongoing acquisitions. When precise registration between sessions is difficult (e.g. due to distortions), the acquisition of distortion-matched structural images [132,133] may be an alternative if fast within-session processing of structural reference data is possible.

On the technical side, real-time fMRI requires very fast processing of the acquired data with guaranteed processing times [38]. High-resolution acquisitions typically result in large amounts of data, which pose challenges to image reconstruction, transfer and analysis [134] that are further exacerbated by the requirements for high-quality registration and spatial processing.

In general, sub-millimetre acquisition is not prohibitive to real-time reconstruction and analysis [84]. However, processing times can vary widely depending on the actual sequence and reconstruction algorithm being used, the total acquisition volume and analysis steps. Also, certain types of analysis methods are incompatible with real-time analysis, e.g. non-causal filters or computationally expensive optimization algorithms.

Practically, if the reconstruction pipeline provided by the vendor is posing limits, it is often possible to introduce custom-made components [135,136]. An innovative approach to speed up image reconstruction for real-time applications like neurofeedback could be the use of an ROI-targeted partial reconstruction [137].

Additional processing steps discussed above to improve specificity and sensitivity, including multi-echo fitting, denoising algorithms or the application of biophysical models would pose further demands. If optimization of software and hardware components is not sufficient, a trade-off between processing benefits and costs of individual components needs to be made.

### (b) Can control of mesoscopic brain activity be learned by neurofeedback?

In a closed-loop fMRI study that measures brain activity at mesoscopic resolution, the typical goal is to manipulate this activity in some way. For example, in the context of neurofeedback at mesoscopic resolution, participants will learn to regulate activity within a given layer of an ROI. However, the effectiveness of this approach relies on whether the regulation of brain activity and its learning also operates at mesoscopic resolution—a question that remains empirical.

**Figure 3.**
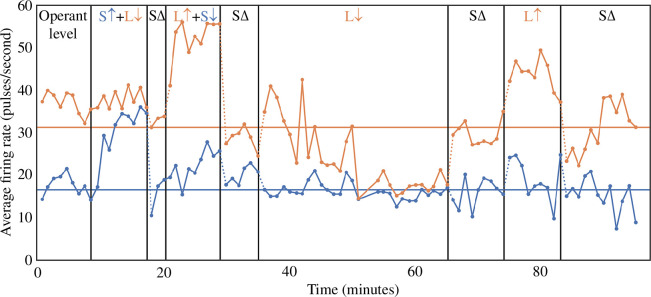
Differential operant conditioning of two independent cortical units (modified with permission from Fetz & Baker [139]). Average firing rates of two isolated units in the monkey motor cortex, a smaller one (S, bottom curve in blue) and a larger one (L, top curve in orange), throughout an operant conditioning experiment are shown. Using specific feedback signals calculated from these units, the monkey learned to differentially up- ( ↑ ) and downregulate ( ↓ ) their activity. During periods labelled with SΔ, no feedback was shown.

There is a long line of research in BCI on volitional control of neuronal activity, with a focus on the motor cortex. Already in 1969, Fetz showed that monkeys could be conditioned to control the electrically recorded activity of individual neurons in the precentral cortex [138]. Figure 3 shows how in a subsequent study [139] a monkey learned to differentially control the activity of two individual neurons. Since then, similar results have been found in the prefrontal [140] and primary visual cortex [141], and even in human visual and association and memory-related areas [142,143].

This control can often be highly specific [143], but not always. Fetz & Baker [139] found that for many but not all studied neighbouring neurons, activity was highly correlated. Furthermore, there are indications that the configuration of neuronal activity affects learning. Learning to control neurons in the parietal reach areas of monkeys was constrained by the already present movement repertoire [144]. Sadtler *et al*. [145] found that the manifold of ongoing neuronal activity constrained what kind of response patterns can be learned. Fisac & Chase [146] found that varying the sensory context by simply visually rotating the biofeedback display affected the volitional control of neurons in the primary motor cortex M1.

There is evidence from decoded neurofeedback (DecNef, [69,147]) at conventional 3 T resolution that may support this view. Shibata *et al*. [69] trained a decoder on orientation responses in V1. Then, without the presentation of oriented stimuli, participants were provided with the ongoing decoder output as feedback. Unaware of the nature of the feedback signal, participants succeeded in increasing the occurrence of brain patterns associated with the decoded orientation and even showed perceptual learning effects. How was it possible to implicitly induce neuronal activity that is thought to occur in sub-millimetre wide orientation columns from feedback signals computed at the level of 3 × 3 × 3.5 mm^3^ voxels? To explain these results Shibata *et al*. [148] proposed a model in which neuronal activity included in the low-dimensional spontaneous neuronal activity of a target region is reinforced.

Together these results suggest that neuronal control in principle is possible as fine grained and specific as at the level of individual neurons. At the same time, results also point to different constraints. Learning is much easier if the activity pattern already belongs to the ‘repertoire’ that is reflected in ongoing activity or responses caused by voluntary actions and cognitive strategies. It has been suggested that prolonging training times and doses could induce changes not possible with less intensive training [143,145,149]. If true, this might reflect how different types of neurofeedback control may be learned [150].

There is limited knowledge about whether precise volitional control is possible in all cortical areas and can act on all types of neurons, and whether there are any laminar constraints. Again, this may depend on the employed mechanism. For example, laminar activation patterns that are associated with some experimental condition like attention or working memory should be easily induced by simply engaging in such a cognitive strategy or behaviour. However, it is less clear whether arbitrary laminar control through implicit operant conditioning as demonstrated at conventional resolutions [69] is possible.

Although high-resolution fMRI can in principle resolve signals at a very fine scale, it is not clear whether we can specifically measure the ‘right’ activity in the context of neurofeedback learning. Associative learning involves three types of signals, the discriminative stimulus that later becomes the control signal, the target response and the reinforcement. All three are associated with neuronal activity. The control signal can be regarded as a top-down input into a brain region, mainly associated with the synaptic and dendritic activity. Neely *et al*. [141] speculated that this is reflected in increased local field potential power in the alpha-band that they observed prior to rewarded target responses. Within the context of laminar connectivity patterns, it is plausible that these control signals follow specified laminar patterns independent of the response. The target response, on the other hand, originates within that brain region and is associated, at least initially, with spiking activity. Lastly, the reinforcement signal is likely a spatially unspecific effect of neuromodulation. The problem with learning to control the response signal in a spatially specific manner is that all three types of signals can contribute to the fed back fMRI signal. However, due to their different nature, they may do so in very different ways [151]. Consequently, it may be much easier to control activity in which the control signal coincides with the target response. Note, however, that it may be possible to disentangle some of these signals through experimental design, e.g. intermittent feedback could be used to move the reinforcement signal towards the end of a block.

The previous points lay out that whether and how voluntary control of laminar activity can be learned may tell us something about the nature of cognitive control and the functioning of the cortex and brain in general. Exploring the feasibility of laminar-specific neurofeedback approaches provides an opportunity to contribute to this topic.

## 5. Conclusions

Closed-loop fMRI at mesoscopic resolution allows for targeting mesoscopic structure and fine-grained representations, probes cortical function and offers a window into mechanisms of cortical control. Moreover, mesoscopic closed-loop fMRI as well as non-real-time functional and structural high-resolution MRI may help us to understand mechanisms of neurofeedback learning and its long-term effects.

Mesoscopic closed-loop fMRI has not been implemented yet and comes with significant challenges, most notably low SNR, and unknown spatial resolution of neuronal control. As such, further progress in MR acquisition techniques, in the understanding of variance components and of neuronal control, will all be beneficial to that quest. However, we estimate that SNR may not be a limiting factor and assess that control at the mesoscopic scale is feasible. In light of this, we advocate for the pursuit of mesoscopic closed-loop fMRI, believing that the current juncture presents an opportune moment to explore this frontier.

## Data Availability

Electronic supplementary material is available online [152].
